# Evaluation of  interfraction setup variations for  postmastectomy radiation therapy using EPID‐based *in vivo* dosimetry

**DOI:** 10.1002/acm2.12712

**Published:** 2019-09-21

**Authors:** Shengwei Kang, Jie Li, Jiabao Ma, Wei Zhang, Xiongfei Liao, Hou Qing, Tingqiang Tan, Xin Xin, Bin Tang, Angelo Piermattei, Lucia Clara Orlandini

**Affiliations:** ^1^ Key Laboratory of Radiation Physics and Technology Institute of Nuclear Science and Technology Sichuan University Chengdu China; ^2^ Department of Radiation Oncology Radiation Oncology Key Laboratory of Sichuan Province Sichuan Cancer Hospital & Institute Chengdu China; ^3^ Institute of Nuclear Science and Technology Sichuan University Chengdu China; ^4^ UOC Fisica Sanitaria Fondazione Policlinico Universitario Agostino Gemelli Rome Italy

**Keywords:** breast radiotherapy, *in vivo* dosimetry, volumetric modulated arc therapy

## Abstract

Postmastectomy radiation therapy is technically difficult and can be considered one of the most complex techniques concerning patient setup reproducibility. Slight patient setup variations — particularly when high‐conformal treatment techniques are used — can adversely affect the accuracy of the delivered dose and the patient outcome. This research aims to investigate the inter‐fraction setup variations occurring in two different scenarios of clinical practice: at the reference and at the current patient setups, when an image‐guided system is used or not used, respectively. The results were used with the secondary aim of assessing the robustness of the patient setup procedure in use. Forty eight patients treated with volumetric modulated arc and intensity modulated therapies were included in this study. EPID‐based *in vivo* dosimetry (IVD) was performed at the reference setup concomitantly with the weekly cone beam computed tomography acquisition and during the daily current setup. Three indices were analyzed: the ratio R between the reconstructed and planned isocenter doses, γ% and the mean value of γ from a transit dosimetry based on a two‐dimensional γ‐analysis of the electronic portal images using 5% and 5 mm as dose difference and distance to agreement gamma criteria; they were considered in tolerance if R was within 5%, γ% > 90% and $\gamma_{\text{mean}}$ < 0.4. One thousand and sixteen EPID‐based IVD were analyzed and 6.3% resulted out of the tolerance level. Setup errors represented the main cause of this off tolerance with an occurrence rate of 72.2%. The percentage of results out of tolerance obtained at the current setup was three times greater (9.5% vs 3.1%) than the one obtained at the reference setup, indicating weaknesses in the setup procedure. This study highlights an EPID‐based IVD system's utility in the radiotherapy routine as part of the patient’s treatment quality controls and to optimize (or confirm) the performed setup procedures’ accuracy.

## 1 INTRODUCTION

Postmastectomy radiation therapy (PMRT) is technically difficult, given the complexity of the target volume and its proximity to critical structures, including the heart, lung, brachial plexus, and contralateral breast.^1^, ^2^, ^3^ More advanced techniques like intensity modulated radiation therapy (IMRT) or volumetric modulated arc therapy (VMAT) can achieve highly conformal dose distributions with improved target volume coverage and sparing of normal tissues compared to conventional techniques. These techniques have the potential to improve treatment outcomes for PMRT and significantly reduce the dose to the heart and the ipsilateral lung.^4^, ^5^, ^6^, ^7^ Nevertheless, uncertainties related to interfraction positioning may lead to inaccuracies in the dose delivered; due to the steepness of the dose‐effect curves, the efficacy of IMRT and VMAT can be limited and the patient outcomes for both local tumor control and normal tissue complications can be affected. As previously reported,^8^, ^9^, ^10^ dose differences of breast treatment in the supine position can be correlated with the patient setup. These errors cannot be detected by pretreatment verification or through accurate quality control of the connected machines and medical devices.^11^, ^12^ The notion has gained ground that these techniques only benefit patients when a good imaging and patient positioning technique is available, suggesting a combined IMRT and image‐guided radiation therapy approach.^13^, ^14^ Cone beam computed tomography (CBCT) scans can be considered a gold standard to assess interfraction uncertainties for many radiotherapy treatments^15^, ^16^ including treatments in the breast area^17^; Jain et al.^18^ registered interfractional systematic (random) setup errors of 5.7 (3.9), 2.8 (3.5), 2.3 (3.2) mm in the lateral, vertical and longitudinal directions significantly affecting the target dose homogeneity (1.8% target received > 105% of the planned mean dose).

Daily acquisition of the treatment images and immediate online correction can reduce the patient setup error’s impact, but it increases department workload, with an unavoidable added dose to the patient that should be considered in the treatment plan.^19^ Three‐dimensional (3D)‐surface imaging systems are a valid tool able to control patient positioning throughout treatment delivery. They were found to be valuable for reducing errors when comparing with patient alignment from skin marks.^20^ Moreover, their technical accuracy has been shown to be quite high,^21^, ^22^ nevertheless their suitability for clinical application is increased when combined with CBCT.^23^

IVD is the last control within the radiotherapy workflow as it is performed during the treatment delivery; it can detect whether the dose delivered to the patient is within the tolerance level and whether the treatment is dosimetrically reproducible. These peculiarities in addition to IVD's capacity to avoid severe accidents distinguish it with respect to image‐ and surface‐guided radiotherapy systems. For these reasons it has been recommended by several international organizations^24^, ^25^ and has become mandatory in some western countries.^26^, ^27^ Moreover, it is widely used in Europe to evaluate the interfractional variations in dose delivery and patient setup for many treatment regions; many studies have validated the technique^28^, ^29^ and presented the results obtained in the clinical practice.^8^, ^30^, ^31^ Mijnheer et al.^32^, analyzed with EPID‐based IVD more than 15 000 plans on different treatment sites, and found that more than 30% exceeded the alert criteria, attributing most of the errors to deviations from the routine clinical procedure and to anatomical changes. This study aims to investigate the robustness of the patient setup procedure in use for PMRT when the CBCT considered the gold standard for the patient setup, is not used daily. EPID‐based IVD was used to evaluate inter‐fraction setup variations occurring in two different scenarios of the radiotherapy routine identified as the reference setup, immediately after the CBCT, and the current setup performed without image guidance. The percentages of electronic portali maging device (EPID) ‐based IVD out of the tolerance level (OTL) registered, were compared and analyzed. The results obtained in the different scenarios were used to identify and adjust weak rings of the overall radiotherapy process.

## 2 MATERIALS AND METHODS

### 2.1 Patients and treatment workflow

A total of 48 breast cancer patients who underwent IMRT (16 patients) or VMAT (32 patients) between September 2017 and March 2018 were included in this study. All patients were immobilized in the supine position using a WingSTEP breast board with head holder and a KneeSTEP knee support (Elekta, Stockholm, Sweden), and they received 50 Gy in 25 fractions in 5 weeks. A sheet of water‐equivalent bolus (ρ = 1 g·cm^−3^) with dimensions of 28 × 28 × 1 cm^3^ was placed over the skin in correspondence with the treatment field  for each radiotherapy session; as almost institutions reported^33^ the chest bolus was used to maximize the radiation dose for the chest‐wall surface and to decrease the risk of local recurrence. Patients underwent a computed tomography (CT) scan with a slice thickness of 3 mm with the bolus sheet in position. CT datasets were imported into Pinnacle ^3TM^ Version 9.10 (Philips Medical Systems, Eindhoven, the Netherlands) treatment planning system. Six Megavolts photon beams of Synergy or Axesse linacs (Elekta, Stockholm, Sweden), available in the department, were used for the treatments. Experienced radiation oncologists conducted the target and organs at risk delineation according to the breast cancer atlas for the radiation therapy planning consensus definitions of the Radiation Therapy Oncology Group.^34^, ^35^ The plan consisted of one or two arcs for the VMAT technique, whereas five beams were delivered via a step‐and‐shoot technique for IMRT. A patient pretreatment verification was performed using an irradiating MatriXX Evolution two‐dimensional (2D) array (IBA Dosimetry, Schwarzenbruck, Germany). Measured and calculated planar dose distributions were compared with the gamma index method using a γ passing rate greater than 90%, with 3 mm distance to agreement and 3% dose difference and a 10% dose threshold.^36^, ^37^ A CBCT was acquired for each patient at the first treatment fraction and then once a week. The couch was moved into the correct position after the CBCT alignment process; however, the maximum accepted displacement was ±5 mm in any one of the x, y, or z directions^38^, ^39^; if higher displacements were required, the patient was aligned again and the CBCT repeated; in case of a persisting error, a consultation with the radiation oncologist for the management of this treatment was scheduled**.** This study was reviewed and approved by the Ethics Committee of Sichuan Cancer Hospital in April 2017.

The daily patient setup, named current setup, comprises the patient positioning on the immobilization device, alignment of the lasers with patient’s CT reference tattoos, and translation of the treatment couch following the treatment planning indications, to align the machine and the treatment isocenters. The daily patient setup followed by the CBCT scan and successive positioning adjustments was named reference setup.

### 2.2 EPID‐based IVD

An EPID‐based IVD was scheduled for each patient twice a week: during the reference and the current setup. A portal image was acquired for each beam with the portal imaging system iViewGT a‐Si panels (Elekta, Crawley, United Kingdom), and it was imported into SOFTDISO version 1.24 EPID‐based IVD software (Best Medical Italy, Chianciano, Italy).^40^, ^41^ Considering five beams for an IMRT treatment and one or two arcs for a VMAT treatment, at the end of the course of radiotherapy, 50 EPID images for each IMRT patient and 10–20 EPID images for each VMAT patient were acquired. SOFTDISO uses a dosimetric method and provides for each IVD test: the ratio R between the reconstructed (*D*_iso_) and planned (*D*_tps_) isocenter doses (R = *D*_iso_/ *D*_tps_) and the γ‐analysis obtained comparing the signal between the first EPID image (reference image obtained at the reference fraction) and the subsequent images acquired during the treatment course by the SOFTDISO are reported in literature.^40^, ^41^, ^42^ The ratio R represents the accuracy of the dose delivered at the isocenter point. The gamma analysis supplies a transit dosimetry to verify the treatment reproducibility, which can be affected by the patient setup, linac output factor variations, beam interruptions, dose calculations, and the presence of patient morphological changes. The ratio R between the reconstructed and planned isocenter doses is considered in tolerance when 0.95 ≤ *R* ≤ 1.05 considering the difficulty in chest wall dosimetry and the statistical propagation of the errors for R (uncertainties of *D*_iso_ estimated in 4% in inhomogeneous tissues, uncertainties in *D*_tps_ within 3%).^43^, ^44^ The global γ‐analysis adopted two gamma criteria: (a) the EPID percentage signal agreement, Δ*S*%, and (b) the distance to agreement, Δ*d* (mm). We adopted as pass criteria 5% and 5 mm. The current choice of pass‐fail criteria aligns with previous literature data^30^, ^45^ and is based on our experience with 2D in vivo dose verification of IMRT‐VMAT using gamma evaluation since the start of routine clinical implementation in 2016.^9^ Δ*d* = 5 mm is also the maximum displacement value acceptable in clinical practice by the radiation oncologists, while the Δ*S* was defined by considering the presence of dose gradients (interface lung‐PTV) and mobility of the irradiated organs (breath). Two tolerance levels were fixed: (a) the percentage γ‐index, γ% ≥ 90.0% (i.e., the number of points with γ<1 must be greater than 90.0%), and (b) the mean γ value, $\gamma_{\mathrm{mean}}$ ≤ 0.4. Therefore, within the EPID irradiated area, a maximum of 10% of the points in disagreement was considered acceptable; moreover, the distribution of the γ values characterized by a mean γ < 0.4 is an indicator of the weight of the discrepancy. An IVD test warning started when even one of the three indices resulted OTL. IVD tests out of tolerance — caused by acquisition errors or due to a lack of sensitivity of the system (predicted dose lower than 5 cGy) — were excluded from the results.

### 2.3 Effectiveness of EPID‐based IVD in detecting errors

The effectiveness of EPID‐based IVD in detecting setup errors was verified for simple geometries with a thorax phantom. CT dataset with a slice thickness of 3 mm of an inhomogeneous anthropomorphic (Alderson) phantom was acquired and imported into Pinnacle treatment planning system. A 6 MV photon beam of Elekta Axesse linac used for the irradiation of the patients included in this study was selected for the treatment planning. The clinical target volume (CTV) and organs at risk were delineated following the same guidelines used for the patients, while the planning target volume (PTV) was obtained with a 3 mm isotropic expansion of the CTV. A VMAT and an IMRT treatment reference plan were optimized for left breast  postmastectomy irradiation with a prescription dose of 50 Gy according to the clinical protocol of our center. Eighteen setup perturbations were introduced into the reference treatment plans, shifting the isocenter (relative to the phantom) in the anterior, posterior, superior, inferior, left and right directions of 3, 5, and 10 mm; **t**he corresponding VMAT and IMRT perturbed plans were recalculated. Absolute difference of the dose volume histogram (DVH) endpoints between the reference and perturbed plans were used to evaluate the clinical impact of these perturbations on the plan dosimetry. Particularly, the dose received by 95 and 98% of CTV (D95 and D98, respectively), the volume of the lung receiving 20 Gy (V20), and the heart mean dose (D_mean_) were used. The perturbations were reproduced at the linac, and EPID‐based IVD was performed applying the same criteria and thresholds used for the clinical study.

Three other type of perturbations were simulated and evaluated with EPID‐based IVD: (a) a sheet of polystyrene (ρ = 0.05 g/cm^3^) with dimensions of 10 × 10 × 0.7 cm^3^ was placed between the bolus and phantom surface in correspondence with the treatment field to simulate an unwanted air gap; the polystyrene sheet was aligned with the center of the bolus sheet and then with its edges (b) the bolus was moved 3 cm away from the medial, lateral, inferior and superior borders of the treatment field; and, (c) the phantom was positioned with the treatment field in correspondence with the bar to lock the extension of the treatment couch.

## 3 RESULTS

### 3.1 Clinical practice

For each pre‐treatment plan verification, the gamma analysis of the measured and calculated planar dose distributions was acceptable with a mean γ% of 94.0% (range 93.1%—100%).

**Figure 1:**
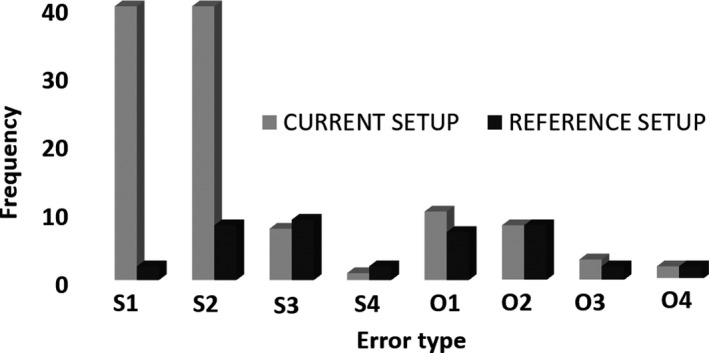
The distribution of interfractional variations for different types of error at the reference vs. current setups. s1: Patient positioning; s2: Imperfect bolus material placement; s3: Use of a bolus with different flexibility at the CT and delivery; s4: Unexpected object in the beam o1/o2: Isocenter in high/low‐dose gradient; o3: Treatment planning: dose calculation grid; o4: Density override of a wire used to define the field border.

One thousand one hundred and ninety EPID‐based IVD checks were scheduled and acquired. However, due to errors during the acquisition process, around 15% of these tests were excluded from the results. Lack of synchronization between SOFTDISO and the beam delivery and wrong (and not realignable) positioning of the EPID were the leading causes of these errors. Table 1 shows the results of EPID‐based IVD for IMRT and VMAT treatments and the current and reference setups. From the results obtained 6.3% (corresponding to a total of 64) of the overall EPID‐based IVD analyzed, resulted OTL. The percentage of OTL registered during the current setup was three times greater (9.5% vs 3.1%) than the one registered at the reference setup. The errors that can contribute to an EPID‐based IVD OTL, and the frequency of their occurrence for IMRT, VMAT, current and reference setups, are reported in Table 2. The errors were divided into *setup errors* and *other errors*. *Setup errors* consisted of errors due to the patient positioning including the positioning within the breast support, the chest bolus positioning, and the accidental presence of attenuators; *other errors* included the errors associated with the treatment planning (isocenter in low‐dose area or high‐dose gradient; accuracy of the dose grid, etc.) and changes in the patient anatomy. More than one error can be associated with a single EPID‐based IVD OTL, leading to a total of 151 errors associated with the 64 EPID‐based IVD OTL. The *setup errors* represented the main cause of the overall EPID‐based IVD OTL, with an occurrence rate of 72.2% (109 out of 151); this rate increased to 80.3% (90 out of 112) when only the current setup is considered. At the reference setup, the frequency of *setup errors* and *other errors* was comparable (19 vs 20 respectively). Figure 1 portrays the distribution of inter‐fractional variations for different types of errors at the reference vs. current setups.

**Table 1:** EPID‐based *in vivo* dosimetry (IVD) for intensity modulated radiation theraphy (IMRT) and volumetric modulated arc therapy (VMAT) treatments, current and reference setups.

EPID‐based IVD	Total	IMRT	VMAT	Current setup	Reference setup
Acquired (number)	1190	800	390	594	596
Discarded (number)	174	116	58	91	83
Analyzed (number)	1016	684	332	503	513
Out of tolerance (number)	64	44	20	48	16
Out of tolerance (%)	6.3	6.4	6.0	9.5	3.1

**Table 2:** Error types associated with EPID‐based *in vivo* dosimetry out of the tolerance level and frequency of their occurrence.

	IMRT	VMAT	Current setup	Reference setup
Setup errors				
Patient positioning	25	17	40	2
Imperfect bolus sheet positioning	28	20	40	8
Bolus with different flexibility (at CT scan vs linac)	16	0	9	7
Unexpected object in the beam	3	0	1	2
Total number	109		90	19
Other errors				
Isocenter in low‐dose area	10	6	8	8
Isocenter in high‐dose gradient	15	2	9	8
Treatment planning: dose calculation grid not including a part of the breast‐support blocking system	5	0	3	2
Anatomy change	0	0	0	0
Treatment planning: density override on field border wire is forgotten	0	4	2	2
Total number	42		22	20
Total	151		112	39

IMRT, intensity modulated radiation therapy; VMAT, volumetric modulated arc therapy.

**Figure 2:**
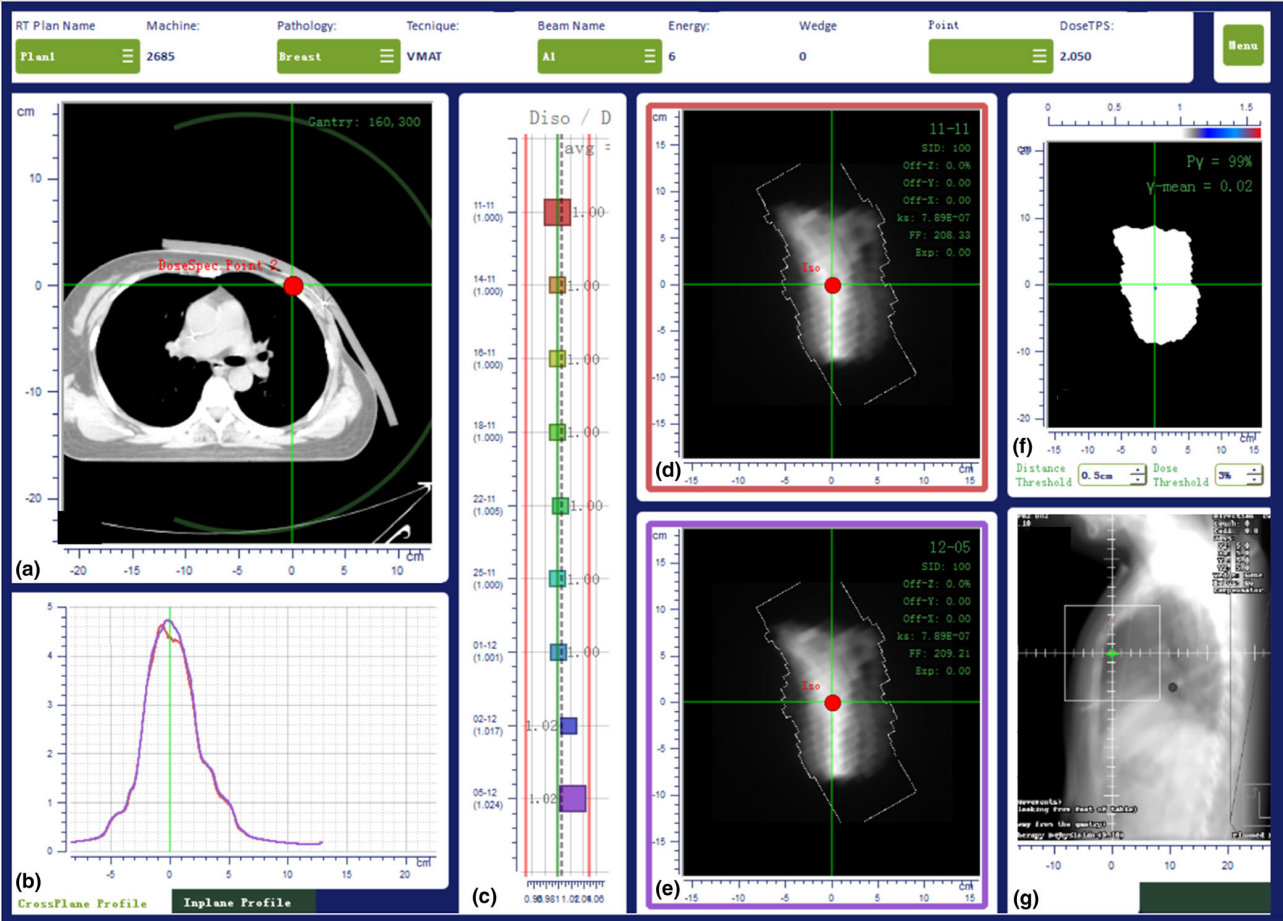
The result of interfractional variation using the EPID‐based IVD. (a) patient CT and planned isocenter, (b) signal profiles and (c) ratio between planned and daily image, (d) reference and (e) daily IVD image, (f) map of points with γ > 1, and (g) map of points with γ > 1 on the sagittal digital reconstructed radiography.

The causes of an EPID‐based IVD OTL were identified with an analysis of the overall data available: SOFTDISO indicators, patient, and treatment parameters. In Fig. 2, the screen as it appears after the delivery of a VMAT treatment. The different panels provide useful information to understand the possible reasons for an OTL. In particular, patient CT and the position of the planned isocenter [Fig. 2(a)], the inline and crossline signal profiles of the EPID images acquired for different fractions [Fig. 2(b)], the R ratio [Fig. 2(c)] obtained between the reference and daily image [Figs. 2(c) and 2(d)], the gamma analysis results and the map of the points with *γ* > 1 over the digital reconstructed radiography [Figs. 2(f) and 2(g)]. These tools of comparison alone are unable to discern the causes of every type of error. However, adding the information of the last CBCT performed, the treatment parameters, on‐site verification of the patient positioning, and the experience of the therapist and medical physicist, generally enable the identification of the cause of OTL. Within the *setup errors* we identified: (a) errors in the alignment of the treatment plan and machine isocenter (γ indices OTL, visual inspection), positioning errors, in particular errors due to an incorrect arm positioning within the breast board (R ratio and γ indices OTL, visual inspection), positioning of the breast support (and therefore of the patient) in correspondence with the connection bar between the treatment couch and its extension (γ indices OTL, visual inspection); (b) incorrect positioning of the bolus sheet (γ indices OTL, visual inspection); (c) bolus with different flexibility used at the planning CT and at the linac (R ratio and γ indices OTL, CBCT, visual inspection); and (d) unexpected object in the beam (γ indices OTL). *Other errors* were registered: in particular, the isocenter in the low‐dose area or high‐dose gradient (R ratio OTL; treatment planning data); the treatment planning calculation grid which did not include a part of the blocking system (γ indices OTL); treatment planning); and density override wire is forgotten (γ indices, last CBCT; treatment planning)**.** No errors due to anatomy change were registered in this cohort of patients receiving radiotherapy between two and six months after surgery depending on the chemotherapy schedule. The causes of EPID‐based IVD OTL were identified and no replanning procedure was required.

The percentage of patients (P%) ending the treatment with the mean values of the indices $\bar{R}$, $\bar{\gamma }$%, ${\bar{\gamma }}_{\mathrm{mean}}$ within the tolerance level and the percentage of the tests (T%) with R, γ%, $\gamma_{\mathrm{mean}}$ indices within the tolerance level are displayed in Table 3. P% was found to be between 96.0% and 100%, while T% varied between 92.0% and 95.8%.

**Table 3:** Percentage of patients and in vivo dosimetry tests with indices within the tolerance level.

Technique	Patients	Tests	P% ($\bar{R}$)^a^	P% ($\bar{\gamma }$%)^b^	P%($\bar{\gamma }$ *_mean_*)^c^	T%(*R*)^d^	T%(γ%)^e^	T%($\gamma_{\mathrm{mean}}$)^f^
IMRT	16	684	100	100	99.8	92.0	94.1	95.8
VMAT	32	332	100	97.4	96.0	92.0	94.2	93.1

IMRT, intensity modulated radiation therapy; VMAT, volumetric modulated arc therapy.

a Percentage of patients resulting with $\bar{R}$ index within the tolerance level.

b Percentage of patients resulting with $\bar{\gamma }$% index within the tolerance level.

c Percentage of patients resulting with $\bar{\gamma }$ *_mean_* index within the tolerance level.

d Percentage of tests resulting with *R* index within the tolerance level.

e Percentage of tests resulting with γ% index within the tolerance level.

f Percentage of tests resulting with γ *_mean_* index within the tolerance level.

### 3.2 Phantom study

Table 4 shows the results of EPID‐based IVD for the 18 perturbed VMAT and IMRT plans.

**Table 4:** Phantom study: dose volume histogram (DVH) dosimetric parameters variations and associated EPID‐based *in vivo* dosimetry (IVD) for plans perturbed with setup errors.

Perturbation			Perturbed vs reference plan absolute difference				EPID‐based IVD indices	
Isocenter shift direction; cm;	dI^a^	Plan	CTV, Δ D95(Gy)	CTV, Δ D98 (Gy)	Lung Δ V20 (%)	Heart Δ D_mean_ (Gy)	γ%; $\gamma_{\mathrm{mean}}$	R
Inferior; 0.3	0	VMAT	0.2	0.3	+2.2	+0.2	99.0%;0.34	0.99
	0	IMRT	0.2	0.4	+1.0	−0.1	99.4%;0.20	0.99
Superior; 0.3	0	VMAT	−0.6	−0.7	−1.2	−0.2	98.4%;0.37	1.00
	0	IMRT	−0.2	−0.6	−0.9	−0.1	99.2%;0.01	1.00
Posterior; 0.3	0	VMAT	−0.4	−0.7	+1.6	+0.2	99.0%;0.27	1.00
	0	IMRT	−0.2	−0.6	+1.2	+0.2	99.2%;0.18	1.00
Anterior; 0.3	0	VMAT	−0.5	−0.7	−1.0	−0.2	99.4%;0.33	1.00
	0	IMRT	−0.2	−0.6	−1.1	−0.1	98.2%;0.31	1.00
Left; 0.3; 0	0	VMAT	−0.3	−0.2	−0.2	0.0	100%;0.05	1.00
	0	IMRT	−0.2	−0.3	−0.4	0.0	99.8%;0.07	1.00
Right; 0.3	0	VMAT	−0.2	−0.4	−0.5	0.0	99.8%;0.11	1.00
	0	IMRT	−0.1	−0.5	−0.6	0.0	99.8%;0.10	1.00
Inferior; 0.5	1	VMAT	−1.4	−2.5	+3.0	+0.3	92.1%;0.39	0.97
	1	IMRT	−1.1	−2.3	+3.0	+0.2	92.8%;0.40	0.97
Superior; 0.5	1	VMAT	−2.3	−3.9	−3.1	−0.3	89.5%;0.43	0.96
	1	IMRT	−2.4	−3.8	−1.5	−0.2	89.6%;0.43	0.96
Posterior; 0.5	1	VMAT	−2.2	−3.9	+5.0	+1.5	91.6%;0.43	1.03
	1	IMRT	−1.5	−2.4	+3.5	+1.5	90.6%;0.43	1.03
Anterior; 0.5	2	VMAT	−4.2	−6.7	−5.0	−1.0	87.6%;0.51	0.95
	2	IMRT	−3.5	−4.3	−3.5	−1.0	87.2%;0.49	0.95
Left; 0.5	2	VMAT	−3.2	−4.9	−1.8	−0.5	88.6%;0.50	0.94
	2	IMRT	−3.0	−4.8	−1.5	−0.5	88.6%;0.50	0.96
Right; 0.5	2	VMAT	−4.2	−5.3	−3.1	+0.2	87.9%;0.45	0.95
	2	IMRT	−4.0	−4.8	−2.7	+0.3	87.4%;0.44	0.95
Inferior; 1.0	2	VMAT	−2.8	−4.0	+5.2	+0.3	89.0%;0.46	0.96
	2	IMRT	−2.8	−4.1	+6.0	+0.4	88.6%;0.49	0.95
Superior; 1.0	2	VMAT	−4.2	−6.9	−5.0	−0.5	88.6%;0.50	0.94
	2	IMRT	−3.5	−4.8	−3.5	−0.5	88.6%;0.50	0.96
Posterior; 1.0	2	VMAT	−3.2	−6.9	+6.0	+3.1	86.5%;0.54	1.05
	2	IMRT	−2.8	−5.6	+4.9	+2.5	86.1%;0.53	1.05
Anterior; 1.0	3	VMAT	−12.0	−15.8	−9.8	−2.8	83.6%;0.57	0.94
	3	IMRT	−9.2	−12.9	−5.0	−3.2	81.3%; 0.71	0.93
Left; 1.0	3	VMAT	−6.2	−11.0	+4.9	−0.1	83.4%;0.57	0.93
	3	IMRT	−6.5	−10.9	+5.1	−0.1	83.4%;0.58	0.93
Right; 1.0	3	VMAT	−9.1	−12.9	−6.0	+0.3	82.9%;0.65	0.94
	3	IMRT	−10.0	−13.6	−4.9	+0.4	84.1%;0.65	0.93

IMRT, intensity modulated radiation therapy; VMAT, volumetric modulated arc therapy.

a Dosimetric impact of the perturbation: 0 (ΔD95 < 0.6 Gy, ΔD98 < 0.7 Gy); 1 (1.1 < ΔD95 < 2.4 Gy; 2.3 < ΔD98 < 3.9 Gy); 2 (2.8 < ΔD95 < 4.2; 4.0 < ΔD98 < 6.9 Gy); 3 (ΔD95 > 6.2 Gy; ΔD98> 10.9 Gy).

The gamma indices are well within the tolerance level for perturbations having low impact on the plan dosimetry while they move away from the acceptability threshold when the perturbations worsened the dosimetry of the perturbed plan. Particularly: (a) γ% > 98.2%, $\gamma_{\mathrm{mean}}$ < 0.37 and 0.99 < R < 1.00, for perturbations of 3 mm around the isocenter that slightly affect the dose coverage (CTV ΔD95 < 0.6 Gy, ΔD98 < 0.7 Gy); (b) 89.5% < γ% < 92.8%, 0.39 < $\gamma_{\mathrm{mean}}$ < 0.43 and 0.96 < R < 1.03, for absolute difference ΔD95 and ΔD98 ranging between (1.1–2.4 Gy) and (2.3–3.9 Gy), respectively; (c) 86.1% < γ% < 89.0%, 0.44 < $\gamma_{\mathrm{mean}}$ < 0.54 and 0.94 < R < 1.05, for absolute difference ΔD95 and ΔD98 ranging between (2.8–4.2 Gy) and (4.0–6.9 Gy), respectively; (d)γ% < 84.1%, $\gamma_{\mathrm{mean}}$ > 0.57 and R<0.94 for absolute difference ΔD95 and ΔD98 ranging between (6.2–12.0 Gy) and (10.9–15.8 Gy), respectively. The increasing dosimetric impact of the perturbations as described in points (a), (b), (c), and (d) is reported in Table 4 with values 0, 1, 2, and 3, respectively.

The perturbations simulating the unwanted air gap with the sheet of polystyrene aligned with the center of the bolus sheet and then with its edges, gave mean γ%, $\gamma_{\mathrm{mean}}$ and R of 89.4%, 0.50 and 0.86, respectively for VMAT plans, and 88.6%, 0.47 and 0.87, respectively for IMRT plans.

The perturbations simulating the 3 cm misalignment of the bolus in the medial, lateral, inferior and superior borders of the treatment field gave mean γ%, $\gamma_{\mathrm{mean}}$ and R of 88.0%, 0.62, and 0.94, respectively for VMAT plans, and 88.9%, 0.65 and 0.95, respectively for IMRT plans.

The perturbation obtained with the positioning of the phantom in correspondence bar to lock the table extension, gave γ%, $\gamma_{\mathrm{mean}}$ and R of 90.1%, 0.44 and 0.94, respectively for VMAT plans, and 89.5%, 0.42 and 0.95, respectively for IMRT plans.

## 4 DISCUSSION

Setup errors are a major source of deviations during EPID‐based IVD verification programs.^8^, ^9^, ^30^, ^32^, ^45^, ^46^ If the setup of a patient is irreproducible, large variations in the dose reconstruction will be observed especially when high‐conformal techniques are utilized. Online setup verification using CBCT information improves the results, but the added radiation dose to the patient and the increase of the department workload contribute to the decision to not program it daily. EPID‐based IVD is complementary to CBCT use, and can provide useful information in the procedure optimization process. From the analysis of the EPID‐based IVD, the OTL registered at the current setup (i.e. when CBCT was not performed before the delivery) were three times higher than the ones obtained during the reference setup and were mainly due to setup errors (occurrence rate 80.3%). This increase of inter fraction setup variation registered during the current setup is an important result because it highlights a lack of robustness of the PMRT setup procedure in use in the department. In this case, the different steps of the radiotherapy path (from the scanning CT to the dose delivery) should be the object of an accurate evaluation to identify the weak link in the radiotherapy chain and minimize possible errors.

Our study identified patient positioning errors due to the following: errors in the alignment of the isocenters (machine/treatment plan) step, due to the high probability to make mistakes in calculating the final lateral, longitudinal, and vertical couch’s values; errors in the positioning of the arms within the WingSTEP support, in particular the aperture angle of the arm laying in the breast support, and arm rotation inside the support (open hands with palm upward vs. hands to tighten the support and palm down). Other authors investigated how the high accuracy needed in the patient arm repositioning remains a crucial requirement.^47^ Additional patient positioning errors occurred when the breast support (and therefore of the treatment field) was aligned with the bar to lock the extension of the treatment couch. The bolus resting on the patient's skin may not perfectly fit with the patient’s body shape, which can be concave in the upper lateral part, under the arms; unwanted air gaps under the bolus for photon radiotherapy may lead to an unexpected skin dose and hinder reproducibility of the treatment.^48^ The bolus sheet's use with well‐defined dimensions and flexibility must perfectly adhere to the patient's skin. Some IVD alerts were registered at the beginning of the study due to the use of two different types of bolus during the CT scans and the treatment. The boluses differed in flexibility, which, in one instance, led to the presentation of a difficult (and non‐reproducible) adhesion of a bolus to the body surface of a patient (who presented an accentuated lateral concave body contour).

Some other errors occurred and were corrected during the treatment: the treatment planning dose calculation grid — which did not include a part of the blocking system — was another cause of the error that led to an underdosage for two consecutive fractions for one of the five beams of an IMRT treatment, which had the beam entry exactly in correspondence to the mask blocking system. Several reports^8^, ^32^, ^49^ discussed that the isocenter positions may be located in a high‐dose gradient region or out of the target; therefore, it may not have clinical significance in some cases. This was the case for some IVD tests performed, for which an isocenter translation by approximately 2 mm was responsible for OTL of the R ratio. These errors were figured out with OTL of the R ratio and the gamma analysis indices with gamma criteria dose and distance to agreement 5%–5 mm, respectively. The threshold selected of 5% for the R ratio and γ% > 90% and $\gamma_{\mathrm{mean}}$ < 0.4 were sensitive enough to highlight discrepancies in the delivered treatment, as shown by the results obtained in the clinical practice and in the thorax phantom.

These values of the alert criteria were chosen to match the current clinical practice. Nevertheless, a retrospective recalculation of EPID‐based IVD objects in this study with (3%–3 mm) gamma analysis criteria gave OTL added percentages of 3.1% and 2.9% at the current and reference setups, respectively, bringing the corresponding OTL percentages to 12.6% and 6.0%. These similar OTL added percentages arising in the two different setup scenarios when  stricter criteria were used, show that setup errors were already catched with (5%–5 mm) gamma criteria; nevertheless further improvement of the treatment accuracy can be achieved.

**Figure 3:**
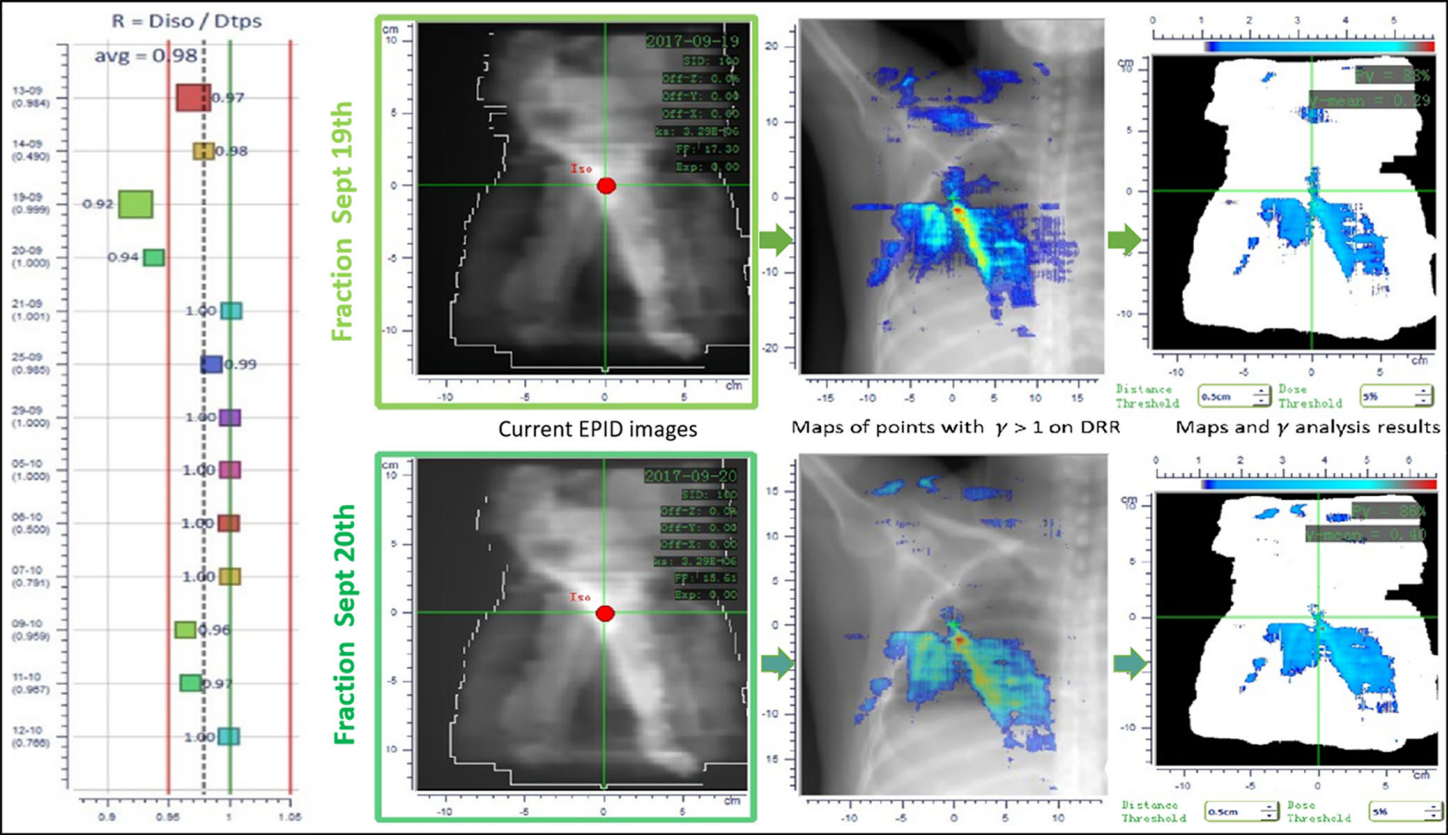
The map of gamma value evaluated using EPID‐based *in vivo* dosimetry. The gamma value indicated out of tolerance during volumetric modulated arc therapy for two fractions.

The accuracy of VMAT and IMRT treatments was investigated using SOFTDISO to find discrepancies in the dose delivered; nevertheless, the EPID image alone cannot provide sufficient additional information to identify the cause of OTL; instead, experience with such difficult fields and additional sources of information must be relied on to find an explanation for the failing beams and arcs. Figure 3 displays the results of IVD checks for a VMAT treatment; the R ratios and the results of the gamma analysis, in particular, fell out of tolerance for two consecutive fractions in the second week of treatment (fractions of 19th and 20th September). In this case, the discrepancy was attributed to a concomitant error of an imperfect bolus placement (identified by the extended region of gamma analysis OTL and by a visual verification) and patient positioning (error in the isocenter, machine/treatment planning) alignment procedure. The causes were identified and corrected before the next session.

The percentage of patients, P%, with the mean value of the indices within the tolerance levels at the end of the course of radiotherapy was greater than 96.0%, regardless of the patient group to which they belonged or the technique used, while T% ranged from 92.0% to 95.8%. This feature of EPID‐based IVD — which enables the timely realignment of the treatment quality indices within the tolerance level — has already been shown in previous studies.^9^, ^30^, ^49^ This indicates that the effect of the corrections was evident and, as expected, every patient treated using the IMRT and VMAT delivery technique ended the treatment with the indices within the tolerance levels.

Some additional techniques have been added to the clinical routine: the isocenter is required to be positioned in the same transversal slice of the tattoos and with only integer displacement from the CT reference setup point; a picture of the patient's arm positioning within the breast support has been added within the record and verification system and is visible at the therapist's console. Moreover, a light field of a zero gantry‐angle beam — which is opened in correspondence with the medial line of the arm and completely opened in the patient longitudinal direction — has been introduced in the daily routine to reduce uncertainties due to the arm opening angle. Treatments requiring the couch extension have been scheduled all together in a defined moment of the day and couch indexes to identify the allowed positions for the breast support docking have been highlighted. Regarding the bolus, a reduction of the margin of acceptability in the bolus positioning has been introduced and only one type of bolus is available at the planning CT and linacs rooms.

This study has some inherent limitations because of its EPID‐based IVD design. The reproducibility of the treatment was verified using 2D measured transit dose distributions and not the 3D delivered dose inside the patient; therefore, we do not have an overall view of how differences that arise can affect the dose volume histogram of the delivered treatment. Nevertheless, Bedford et al.^50^ compared a 3D back‐projection with a 2D forward‐projection EPID dosimetry obtaining a moderate agreement in the results, showing that both methods can contribute to the verification of the dose delivered to the patient.

In our study, we investigated PMRT with the aim to detect setup interfraction variations occurring in the clinical routine in order to refine and optimize the current setup procedure in use in the clinical practice. With the certainty of the consolidated setup procedure for PMRT, the authors intend to go further with an accurate analysis investigating errors highlighted by more stringent thresholds and gamma criteria,^37^, ^51^ and extending the study to other types of radiotherapy treatments.

## 5 CONCLUSIONS

Interfraction setup variations occurring at current setup in the daily clinical routine of PMRT can be higher than the ones registered in concomitance with the CBCT scheduled along the radiotherapy course, pointing out weaknesses of the setup procedure in use.

The study highlights the feasibility and utility of an EPID‐based IVD system in the radiotherapy routine as part of the patient’s treatment quality controls and to optimize (or confirm) the accuracy and reproducibility of the procedures performed.

## CONFLICT OF INTEREST

There is no conflict of interest declared in this article.
